# Comparative Analyses of Gene Co-expression Networks: Implementations and Applications in the Study of Evolution

**DOI:** 10.3389/fgene.2021.695399

**Published:** 2021-08-13

**Authors:** Katie Ovens, B. Frank Eames, Ian McQuillan

**Affiliations:** ^1^Augmented Intelligence & Precision Health Laboratory (AIPHL), Research Institute of the McGill University Health Centre, Montreal, QC, Canada; ^2^Department of Anatomy, Physiology, & Pharmacology, University of Saskatchewan, Saskatoon, SK, Canada; ^3^Department of Computer Science, University of Saskatchewan, Saskatoon, SK, Canada

**Keywords:** gene co-expression networks, network alignment, gene expression, comparative transcriptomics, evolution

## Abstract

Similarities and differences in the associations of biological entities among species can provide us with a better understanding of evolutionary relationships. Often the evolution of new phenotypes results from changes to interactions in pre-existing biological networks and comparing networks across species can identify evidence of conservation or adaptation. Gene co-expression networks (GCNs), constructed from high-throughput gene expression data, can be used to understand evolution and the rise of new phenotypes. The increasing abundance of gene expression data makes GCNs a valuable tool for the study of evolution in non-model organisms. In this paper, we cover motivations for why comparing these networks across species can be valuable for the study of evolution. We also review techniques for comparing GCNs in the context of evolution, including local and global methods of graph alignment. While some protein-protein interaction (PPI) bioinformatic methods can be used to compare co-expression networks, they often disregard highly relevant properties, including the existence of continuous and negative values for edge weights. Also, the lack of comparative datasets in non-model organisms has hindered the study of evolution using PPI networks. We also discuss limitations and challenges associated with cross-species comparison using GCNs, and provide suggestions for utilizing co-expression network alignments as an indispensable tool for evolutionary studies going forward.

## 1. Introduction

Biological systems can be studied as large-scale networks such as gene expression networks, protein-protein interaction (PPI) networks, and metabolic networks (Serin et al., 2016). Comparing these networks is valuable for understanding the relationships between biological entities across different phenotypes and throughout evolution (e.g., diseased vs. healthy, good prognosis vs. bad prognosis, mouse vs. human, etc). Studying how these networks are “re-wired” can provide more insight than studying biological entities as independent units that do not interact with each other. Many methods are available for PPI network analysis and comparison. However, developing a specific PPI network is a challenging task for non-model organisms, which is critical for making evolutionary inferences (Schoenrock et al., 2017). On the other hand, developing gene expression networks is a straightforward task due to publicly available gene expression profiles for model and non-model organisms.

The relationships between genes can be inferred using an organism's transcriptome, which traditionally referred to all messenger RNA (mRNA) molecules expressed, but now describes the full range of RNA transcripts expressed by an organism. The transcriptome is closely tied to an organism's phenotype, such as morphological structure (Olson, 2006); therefore, transcriptomic activity can affect organismal functions. With the advance of high-throughput technologies such as RNA-seq and single-cell RNA-seq, comparative transcriptomics has become useful for tracking gene expression changes that might underlie molecular mechanisms of evolution (Gómez-Picos and Eames, 2015). Gene expression networks make it possible to study coordinated gene expression patterns across various phenotypes and organisms.

Gene co-expression networks (GCNs) represent gene–gene interactions as an undirected graph, where the nodes of the graph represent genes and edges represent the co-expression strength between nodes (Stuart et al., 2003). Although these networks do not contain information about regulation direction, they still allow for the simultaneous analysis of many genes and the potential relationships between them. GCNs can be compared across different tissues, cell types, or species to better understand the coordinated changes in gene-gene interactions (van Dam et al., 2017). Several techniques are currently utilized to make cross-species GCN comparisons, including differential co-expression network analysis methods (Watson, 2006; Langfelder and Horvath, 2008; Tesson et al., 2010; Amar et al., 2013), inter- and intra- modular hub detection (van Dam et al., 2017), and functional annotation transfer (Proost and Mutwil, 2017; Reyes et al., 2017; van Dam et al., 2017).

Comparative analyses of GCNs can be a valuable approach to generate hypotheses and gain insight into the evolution of biological processes using the similarities and differences between the biological interactions across multiple species. Homologous genes, for example, tend to be negatively correlated with molecular evolution rates and co-expression connectivity changes are more likely in genes that are relatively younger in evolutionary history that tend to have low connectivity (Monaco et al., 2015; Wei et al., 2016). Genes with lower connectivity—where fewer edges connect the genes to other genes of the network—also tend to be co-expressed with other young genes (Wei et al., 2016). Gradually, these young genes can become more connected and can potentially become hubs in the network depending on how important they are to functional processes. The comparison of GCNs has also being made by mapping orthologs between species and comparing modules that are associated with particular functional processes (Stuart et al., 2003; Yan et al., 2014). Differential co-expression analysis also detects differences in the co-expressed genes between two conditions, typically diseased and healthy samples (Jiang et al., 2016), but can also compare two species (van Dam et al., 2017; Muley et al., 2020).

In this paper, we focus on using GCN comparisons of species to identify evidence of adaptation and conservation. Network alignment and alignment-free methods can address the lack of knowledge regarding how each node of one network maps to one or more nodes of the other network(s), and identify areas where GCNs are conserved or different (Memišević and Pržulj, 2012). However, several challenges exist when comparing and aligning GCNs, PPI, gene regulatory, metabolic, and ontology networks. Depending on the strategy chosen, the network alignment method can be computationally intractable, requiring heuristics. Further, the best network alignment methods for GCN alignment specifically is unknown.

In section 2, we explain how the general representation of GCNs differs from PPI networks and section 3 covers applications of network alignment to evolutionary studies. In section 4, we discuss the trade-offs between local, global, pairwise, and multiple alignment-based methods in the context of evolutionary studies. Section 5 describes the available tools and methodologies to align GCNs, including common alignment-free methods, while highlighting their shortcomings. In section 6, we provide suggestions for addressing current challenges in comparing GCNs. Finally, section 7 concludes the paper.

## 2. Co-expression Network Representation

There are several ways in which biological networks may be represented graphically, with different methods to represent relationships between nodes. PPI networks typically have edges that have no associated weight. A weighted graph can also be used, where the edge weight can also signify how confident, based on available data or experimentation, one can be that the edge is present (Gitter et al., 2010). This is typically represented as a value between 0 and 1, with 1 being the highest confidence and 0 being the lowest confidence. The associations between proteins can range from direct physical interactions inferred from an experimental method to functional relationships that are predicted on the basis of computational analysis of other known biological data. Some examples of edge weight values include socio-affinity index, which provides a measure of the association between a pair of proteins based on an entire affinity purification-mass spectrometry dataset (Gavin et al., 2006; Rao et al., 2014). The measure is determined by considering when a protein is able to retrieve another when tagged, when two proteins are retrieved by another protein, and the overall frequency of each protein in the dataset. Known interactions from primary databases can also be utilized, including pathway knowledge from databases such as KEGG, measuring the similarity between protein structures, or utilizing gene information such as conserved relationships across multiple genomes to suggest there is a potential possibility of the functional relationships among the proteins encoded by the related genes (Rao et al., 2014).

GCNs are constructed from high-throughput measurements such as microarray and/or RNA-seq. In the context of evolutionary studies, RNA-seq has the added benefit of not being limited to only model organisms that have prior genomic resources, which could allow for comparisons of gene expression across a large number of species at one time (Todd et al., 2016). However, building transcriptomes without a good genomic resource can lead to a less accurate assembly.

GCNs typically use weighted graphs. One of the most common similarity measures used to construct these weights is correlation, an association measure used to estimate the relationships between two variables. Pearson correlation coefficient measures the extent of a linear relationship between variables *x* and *y* and is a preferred and standard way of calculating GCN edge weights. Other measures that are not as common include Spearman correlation, which is based on rank, measuring the extent of a monotonic relationship between *x* and *y*. All correlation coefficients take on values between −1 and 1, where negative values indicate an inverse relationship, such as transcriptional repression. A correlation coefficient is an attractive association measure since it can be easily calculated, allows for calculating significance levels (*p*-values), and the sign (±) allows one to distinguish between positive and negative relationships. For gene network prediction, close relationships have been found between mutual information and correlation-based GCNs. Mutual information is often highly related to the absolute value of the correlation coefficient and when they disagree, the correlation findings appear to be more plausible statistically and biologically (Steuer et al., 2002; Song et al., 2012). Since mutual information requires discrete data, it is not usually preferred over Pearson correlation as it usually leads to loss of signal. One advantage of mutual information is that it captures non-linear relationships, which is not possible with other preferred metrics (Liu, 2017). Simple measures such as these have been found to be among the highest performing for measuring network connectivity and functional inference (Ballouz et al., 2015).

**Figure 1:**
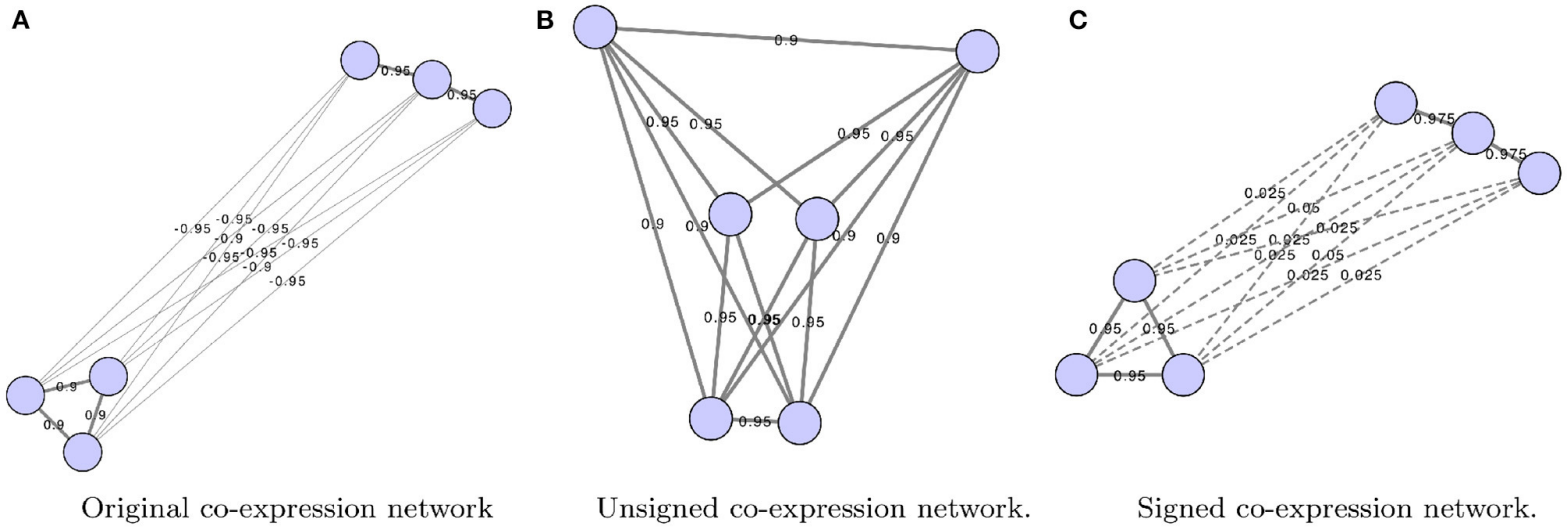
Co-expression networks show the difference between original **(A)**, unsigned **(B)**, and signed gene co-expression networks **(C)**. The original network shows edge weights as calculated using a correlation measure such as Pearson correlation. Networks were constructed using Cytoscape (Shannon et al., 2003).

Some of the more common ways GCNs represent edge weights are shown in Figure 1. First, the edges can be weighted from −1 to 1 using simply the correlation coefficient. Alternatively, edges may be weighted using the absolute value of correlation coefficients, using

(1) $$\begin{array}{l} |cor\left(e_{xc},e_{yc}\right)|, \end{array}$$

where *e*_*xc*_ is the expression of gene *x* in condition *c*. This is referred to as unsigned correlation, and has the effect of mapping both positive and negative correlation toward 1, and no correlation toward 0. Furthermore, correlation can also be transformed to be between 0 and 1 by using the following equation:

(2) $$\begin{array}{l} 0.5+0.5*cor\left(e_{xc},e_{yc}\right), \end{array}$$

This is referred to as signed correlation. A value closer to 0 is a strong negative correlation, a value closer to 1 is a strong positive correlation, and a value of 0.5 indicates no correlation (Langfelder and Horvath, 2008). Although this method retains information regarding negative and positive correlation, typically this method is not used to align networks.

These networks are often thresholded either using a strict cut-off that is applied to filter out non-important edges or the network has a soft threshold applied (Tsaparas et al., 2006; Langfelder and Horvath, 2008; Yan et al., 2014; Monaco et al., 2015). A soft threshold, on the other hand, retains all edges. Each of the edge weights is taken to a power so that when lower weights are taken to the power, the weights are pushed closer to zero. When stronger correlations are taken to the power they are emphasized.

In the following section, we provide motivations as to why utilizing network alignment methods to study evolution may be beneficial.

## 3. Potential of Network Alignment Applications to the Study of Evolution

The alignment of genes and proteins at the network level could be important in order to understand the evolution of their function. Since these networks are functional in nature, the evolution of these networks must be functionally constrained; thus their topology should also be similarly constrained. As such, like with sequence alignment, the alignment of GCNs should reveal information as to the evolutionary history of the GCN. Regions of the GCNs that are relatively constrained suggest the conserved genes serve essential functions. If the connections between genes based on their correlation changes frequently across species, then it is more likely that these genes lack of functional conservation and perhaps even suggests the evolution of new biological functions or modules.

**Figure 2:**
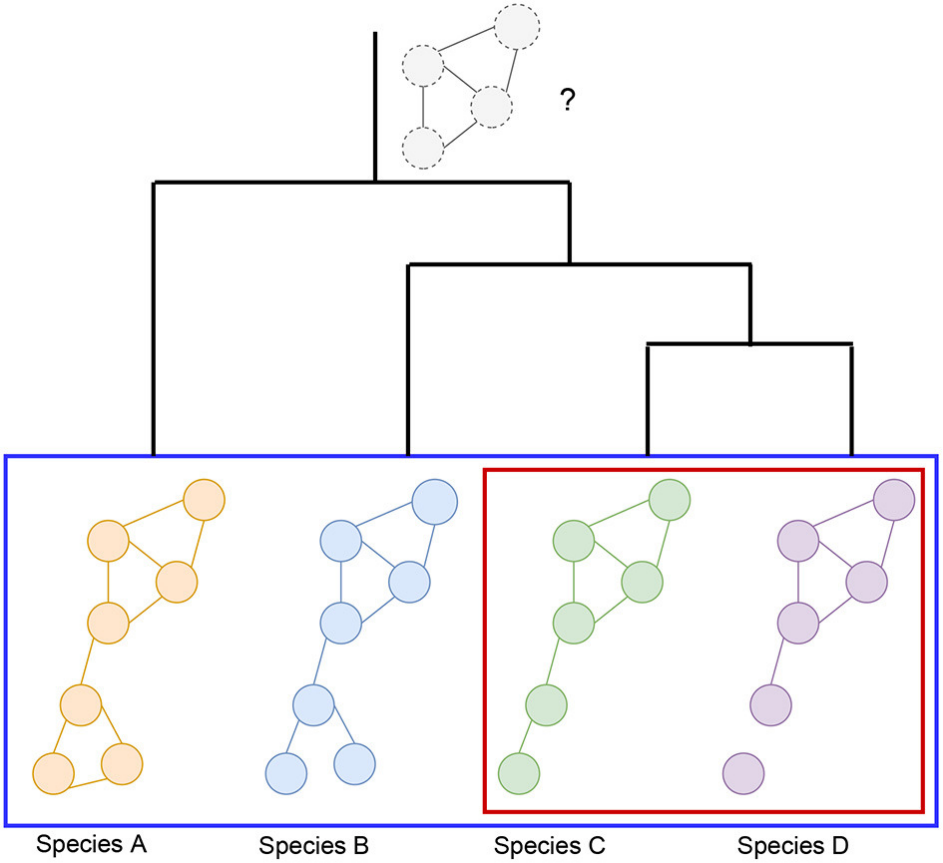
Visualization of the hypothetical changes in a biological network, such as a co-expression network, generated from four different species (species *A, B, C*, and *D*). Different comparisons that can be made include the pairwise comparison between the GCNs of two species (red box), or simultaneous comparisons can be made across many species (blue box). These types of comparisons may also be used to infer ancestral GCNs, which may be inferred as portions of the networks that are more conserved across the species being compared. The known information about phylogeny and gene age can also be utilized in order to reconstruct hypothetical ancestral GCNs, such as the one represented at the root of this phylogenetic tree. It is important to note that the ancestral GCN is completely preserved across these species for the sake of this example, but this is unlikely without any evolutionary changes over time. In this alternative scenario, the relationships between the bottom module nodes would not be preserved, suggesting that perhaps whatever this module of genes was responsible for biologically is no longer important for the later diverged species.

Graph alignment can be used to infer ancestral networks by identifying conserved subnetworks. Ancestral reconstruction is the extrapolation back in time from measured characteristics of species to their common ancestors (see Figure 2). It should be mentioned that there are non-alignment based methods attempting to predict the core nodes and interactions that may have been a part of the network's ancestral state (Simões-Costa and Bronner, 2013). Reconstructing these networks commonly involves the modeling of loss or gain of interactions following gene duplication or losses during long-term evolutionary processes (Conant and Wolfe, 2006; Gu et al., 2019). Others also incorporate substitution rates and mutation data in order to predict what an ancestral network looks like (Beleva Guthrie et al., 2018). However, these methods tend to rely on generative probabilistic models of module evolution to represent the expression modules in each species and assign genes to modules in each extant and ancestral species (Roy et al., 2013; Koch et al., 2017; Liebeskind et al., 2019).

Differential network connectivity and module conservation can be used to identify key drivers of evolutionary change (Madan Babu and Teichmann, 2003; Babu et al., 2004). Currently, interpreting the specific gene expression differences between species and determining the evolutionary significance of these changes is a significant challenge. It is possible for differences in gene expression to evolve neutrally and have little functional consequence. Thus, tools are required that can systematically discern between gene expression changes that are likely to be functionally significant and those that are not. The ability to compare gene expression at the network level can provide a better picture of gene evolution at the systems level. The changes in connectivity between genes can provide indication as to potential functional consequences.

Current automated computational methods to assign functional labels to unstudied genes often involve transferring annotation from orthologs (van Dam et al., 2017). However, since genes can evolve different functions, these transfers would benefit from knowledge of the organization of these genes in networks. From an evolutionary perspective, these networks can be used to identify likely functional orthologs (orthologs that share the same or similar biological role) in species with less information, identify evolutionarily conserved sub-graphs, as well as identify conserved functions.

The following sections will briefly describe work in comparing networks using homology and topology as similarity measures. Most of the specific examples of alignments involve PPI networks as this is the biological data for which most of these methods have been designed. Comprehensive reviews of the many methods or tools available for network alignment are covered in the literature (Clark and Kalita, 2014; Faisal et al., 2015; Elmsallati et al., 2016; Emmert-Streib et al., 2016; Meng et al., 2016b; Guzzi and Milenković, 2017).

## 4. Graph Alignment in Biological Networks

The principle behind biological network alignment is that biologically relevant associations are likely to be observed in different individuals, species, tissues, or conditions whereas false associations are less likely to be repeatedly observed. For example, the conserved genes in terms of both sequence and expression among multiple species are expected to play a key role in biological responses (Stuart et al., 2003). The goal is therefore to align the networks to identify these conserved elements. In order to better understand the application of network alignment to co-expression networks, it is important to consider the techniques used with other types of biological networks such as protein-protein interaction (PPI) networks.

For network alignment, the basic problem is represented as follows: each network is represented as a graph *G*_*i*_, where *G*_*i*_ = (*V*_*i*_, *E*_*i*_) with *V*_*i*_ being a set of nodes and *E*_*i*_ being the set of edges that connect nodes in *V*_*i*_. Some scoring scheme is defined between components of the graphs, and the goal of an alignment between two networks *G*_1_ and *G*_2_ is to map as many nodes and edges in one graph to the nodes and edges (respectively) of the other in such a way that the sum of scores is high. However, there are many factors that can be integrated into the scoring scheme, which will be explored next.

Network alignment strategies can be considered global or local. The goal of local alignment is to find conserved subnetworks in a graph; since multiple local alignments can exist, this means that individual nodes in one graph can have multiple good local alignments. These methods tend to identify subnetworks or communities of related genes. In comparison, global alignment methods typically align every node in one network to a node in another network, attempting to find the one alignment with the maximum amount of similarity (Meng et al., 2016b).

Network alignments can also be independently divided into two categories: uniquely labeled, and unlabeled. For the first, the two graphs have labeled nodes, which could be e.g. gene name (in principle, graphs could have labeled edges as well). A uniquely labeled network has separate labels for each node. In a uniquely labeled alignment, it forces a node to align with the (at most one) similarly-labeled node in the other graph. It should be noted that it is possible to create optimal uniquely labeled alignments in a computationally efficient manner (in polynomial time) (Dickinson et al., 2004). An example of an alignment of uniquely labeled networks maps one-to-one orthologs between species to each other. In contrast, unlabeled alignments would ignore any labels on the graphs, and align based on topological similarity only. An alignment between unlabeled graphs (or ignoring the labels) may in some scenarios be desirable in order to focus on comparing the structure of the graphs. In the context of comparing networks across species to study evolution, comparing network structure without relying on known biological relationships between the genes may be beneficial when aligning networks from organisms that are not model organisms and as such, may lack informative labels. Homologs also may not have the same functions, so it is possible these genes should be aligned with other genes in the networks responsible for similar functions.

Both unlabeled local and global graph alignment are usually computationally intractable to solve optimally. As an example, just the problem of determining whether two graphs are isomorphic (they are the same after renaming nodes and edges) has no known polynomial time algorithm. It is also possible to use both topological and sequence similarity by utilizing a cost function that combines them together. For example, one such cost function is

(3) $$\begin{array}{l} C\left(u_{i},v_{j}\right)=\alpha T\left(u_{i},v_{j}\right)+\left(1-\alpha \right)H\left(u_{i},v_{j}\right), \end{array}$$

where *u*_*i*_ and *v*_*j*_ are nodes in networks *i* and *j*, respectively, and *T* is some topological scoring function representing the similarity of the topological neighborhood of the nodes in their networks; and *H* is a biological scoring function indicating the similarity of the genes at a sequence level. This means that the alignment of orthologs could be considered in the calculation of a score, but it does not necessarily enforce a mapping between these orthologs. This can be thought of as a hybrid of the uniquely labeled and unlabeled approaches. In order to vary how much influence each of the similarities have to the overall score, a parameter α used in Equation (3), which is a fixed value between 0 and 1. If α is chosen to be closer to 0, sequence similarity has more influence on the similarity between nodes; if α is chosen to be closer to 1, topological information has more influence. Some of the newer methods of network alignment also allow for updating this cost function after each iteration of an alignment—after some nodes have already been aligned—which could provide information for the remaining iterations (Neyshabur et al., 2013; Sun et al., 2015; Guzzi and Milenković, 2017).

### 4.1. Biological Similarity in Network Alignment

An important aspect of generating informative network alignments is ensuring the alignments make sense from a biological perspective. Using a measure of biological similarity can aid in generating biologically relevant alignments. These measures of similarity can be accounted for using the *H* function described in section 4. One measure of similarity used between genes in this case is sequence similarity, a traditional method of identifying homology. Scores can be calculated using BLAST between protein or gene sequences with *E*-values less than some threshold, or by identifying the known orthologs between species (Stuart et al., 2003). Some methods use only these calculated BLAST E-values as part of their alignment cost functions, or they can also incorporate a variety of similarity information including sequence, structural, and ontology information (Clark and Kalita, 2014).

Utilizing gene sets or gene set enrichment are other potential strategies for not only evaluating, but also driving the alignment of networks (Kuchaiev and Pržulj, 2011). A gene set refers to a set of genes that have been grouped and annotated with particular functions based on prior biological knowledge. A gene set may be considered enriched when it shows statistically significant and concordant differences between the networks being compared. Gene Ontology (GO) terms are a controlled vocabulary that describes biological properties of gene products, and the Gene Ontology is the organization of these terms that describes their relation to each other. They can also be referred to as gene sets. The terms are organized as a directed acyclic graph (DAG) where each node is a GO term, and each edge is the relationship between the GO terms. One strategy is to determine the semantic similarity between the GO terms for each node across the networks being compared (Shui and Cho, 2016). To do so, the subgraph of GO terms annotating each node of a network is transformed into a vector of information content distance for every GO term pair. A pair of nodes across the networks being compared can then be compared, and a measure of similarity between the nodes is calculated as the Euclidean norm between the distance vector for each node to get a similarity score and determine good alignments between the networks. Another simple method typically used to evaluate an alignment based on GO terms is to calculate the fraction of aligned proteins sharing the same GO terms (Kuchaiev and Pržulj, 2011). The larger the fraction, the more biologically meaningful the alignment. The GO terms can also be weighted based on their frequency or how informative they are (Hayes and Mamano, 2017). Other alignment methods use the number of distinct GO terms that are statistically significantly enriched in the modules identified in each network as a measure of conservation and alignment quality (Kalaev et al., 2008; Faisal et al., 2015).

The current limitations of gene set analysis may hinder the comparison of gene sets enriched between the networks being compared (Maleki et al., 2020). The databases and gene set enrichment analysis methods selected in order to perform the enrichment analysis and drive or evaluate network alignments can have a substantial impact on the results of the enrichment analysis and consequently the alignment (Maleki et al., 2018, 2019a,b). Also, using known annotation to align the networks likely will not be as useful in scenarios when the goal is to align networks in order to transfer annotation from one species to another species with limited annotation. Therefore, incorporating topological information is also useful for guiding network alignments.

### 4.2. Topological Similarity in Biological Network Alignment

Some alignment methods rely on strategies to measure similarity between the topological properties of networks. Common similarities include calculating differences between degrees, clustering coefficients and eccentricities (Kuchaiev et al., 2010; Hashemifar and Xu, 2014), spectral signatures (Singh et al., 2007; Liao et al., 2009; Patro and Kingsford, 2012), and graphlet-degree signatures (Milenković et al., 2010; Memišević and Pržulj, 2012; Malod-Dognin and Pržulj, 2015). For example, alignment could involve aligning graphs based on similarity of neighbors, where two nodes are considered a good match if their neighbors are also good matches.

IsoRank is the original graph alignment method introduced to align PPI networks (Singh et al., 2007), and it has also been used to align GCNs (Liao et al., 2009; Ficklin and Feltus, 2011; Yan et al., 2014). In the original algorithm, the guiding principle was that if two nodes of different networks are aligned, then their neighbors should be aligned as well. It is an application that uses spectral methods, whereby the eigenvalues and the eigenvectors of the adjacency matrix of a graph are invariant with respect to node permutations. Therefore, if two graphs are isomorphic, their adjacency matrices will have the same eigenvalues and eigenvectors (Conte et al., 2004). The proteins are ranked by their total weights based on topological similarities using an iterative spectral clustering algorithm to identify conserved proteins. IsoRank and IsoRankN are capable of aligning 5 and 6 species at most, respectively, due to their exponential time complexity (Hu et al., 2014). Furthermore, handling large networks of more that 10,000 proteins or genesis a challenge (Shih and Parthasarathy, 2012).

### 4.3. Current Directions to Improve Biological Alignment Strategies

Some of the main strategies for improving alignment methods are to (1) combine local and global alignment methods (Meng et al., 2016a; Milano et al., 2019), (2) improve the agreement between topology and homology similarity (Guzzi and Milenković, 2017), (3) consider both node and edge similarities when making alignments (Crawford and Milenković, 2015; Sun et al., 2015; Vijayan et al., 2015), (4) align more than two networks (Kalaev et al., 2008; Flannick et al., 2009; Liao et al., 2009; Vijayan and Milenković, 2017), and (5) combine groups of alignment methods (Malod-Dognin et al., 2017; Manners et al., 2017). The limitations of using either local or global alignment is being addressed with methods that try to find a balance between local and global alignment, which have been shown to be complementary (Meng et al., 2016a). Therefore, it may be beneficial to use both or incorporate features of both alignment methods in a single method. IGLOO, for example, utilizes an already available (interchangeable) local alignment method to make an initial alignment, and then applies a global alignment strategy to improve topological similarity scores (Meng et al., 2016a). As another example, GLAlign initially applies MAGNA++ (a global alignment method) to collect a list of matching nodes and a list of seed nodes generated from biological information. Then Align-MCL (a local alignment method) is used to produce the final alignment (Milano et al., 2019).

The majority of the methods described in section 4 have been tested with and applied to PPI networks. The benefit of using gene expression networks as opposed to PPI networks to study evolution is that the PPI networks available today across a variety of species are largely incomplete. Depending on the species or tissues a researcher wishes to study, it may be difficult to obtain enough PPI information. It is much easier to collect high-throughput sequencing data for many species, which can be used to generate GCNs. The following section describes how GCNs have been compared using network alignment including methods that have been applied or designed specifically with GCNs in mind.

## 5. Alignment and Alignment-Free Methods and Applications to Gene Co-expression Networks

Co-expression networks exhibit many of the same properties as PPI networks. They both tend to have a scale-free structure and have a strong modularity (Carlson et al., 2006). They also both have a number of highly connected nodes that are known as hubs (Gaiteri et al., 2014). However, although many GCNs have been constructed, few PPI network alignment techniques have been utilized for comparing GCNs, especially from eukaryotic organisms. Section 5.1.1 includes a demonstration of WGCNA, which is a commonly used method for module detection in GCNs and can also be utilized to compare networks. Section 5.2 contains a discussion of PPI alignment methods that have been applied to GCNs, methods developed specifically to align GCNs, and a description of their applications. First, section 5.1 describes some methods and applications of comparing GCNs without creating alignments.

### 5.1. Alignment-Free Comparisons of Co-expression Networks

**Figure 3:**
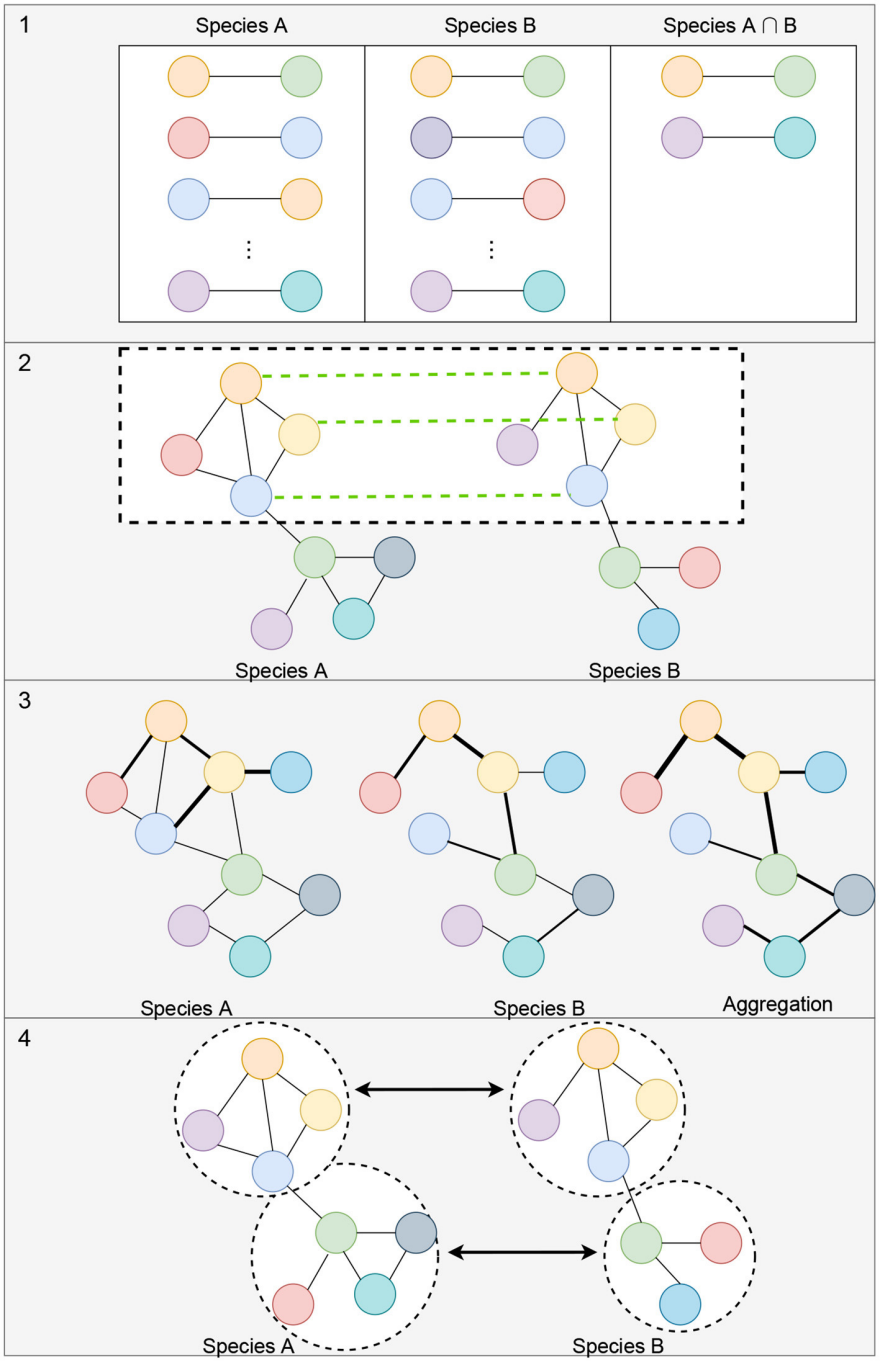
Illustration of alignment-free strategies to measure the similarity between gene co-expression networks. (1) Measuring the topological similarities between networks. This could include identifying the conserved gene-pairs as shown in the illustration or more complex subgraph comparisons such as counting the number of conserved small subgraphs (3–5 nodes), like triangles, 2-stars, 3-stars, squares, or cliques. (2) Representation of methods that utilize orthologous links (shown with green links between the networks) to identify conserved modules of genes. (3) Example of comparison of edge weights or network aggregation where the nodes of the graph are automatically matched up and the edge weights aggregated to obtain a measure of similarity between the networks. (4) Module detection where each module can be compared using network connectivity and density statistics.

Alignment-free network comparisons aims to quantify the similarity between networks using other methods besides network alignment (Mutwil et al., 2011; Netotea et al., 2014; Serin et al., 2016; Tzfadia et al., 2016). These approaches may include measuring the similarity between the topological properties of networks (Tsaparas et al., 2006; Ali et al., 2014; Leal et al., 2014; Monaco et al., 2015; Jardim et al., 2019; Zu et al., 2019), clustering for the identification of conserved modules of genes (Stuart et al., 2003; Gerstein et al., 2014; Yan et al., 2014), and comparison of edge weights or network aggregation for matched orthologs (Jo et al., 2018; Lee et al., 2020). Figure 3 illustrates some of these strategies for measuring the similarity between networks. Since these methods are not designed to (directly) generate a mapping between all of the nodes of the networks, beyond known orthologous relationships, we do not consider them as network alignment methods. However, many of these methods work to match up groups of genes, or clusters, so we discuss these types of methods in section 5.1.1.

#### 5.1.1. Cluster Alignment Methods

Clustering has been utilized to identify evidence of conservation in gene co-expression across vertebrate species (Oldham et al., 2006; Chan et al., 2009; Weber and Hurst, 2011; Gerstein et al., 2014). Many methods designed explicitly for co-expression network comparison generate a mapping between clusters (Yan et al., 2014). These methods link modules of co-expressed genes together based on the known orthology relationships of genes. We refer to these methods as cluster alignment methods.

Yan et al. proposed OrthoClust based on a simulated annealing strategy. OrthoClust aims to discover the optimal assignment of orthologs to modules based on a cost function considering the modularity and known orthologous links between genes within clusters (Yan et al., 2014). They evaluated their method based on a set of 1,288 genes reported to have conserved expression patterns across several species, including worm and fly. These genes were referred to as metagenes and expected to be in aligned clusters. The authors reported that when compared to the alignment method IsoRank, 88% of metagenes were aligned by IsoRank while 81% were grouped in the same clusters by OrthoClust. This observation suggests that PPI network alignment methods could lead to biologically meaningful results for comparing GCNs.

A limitation of most clustering-based approaches is that they assign each gene to a single cluster; however, genes could be involved in different regulatory pathways depending on the conditions they are acting under. Biclustering on the other hand, can be used to simultaneously cluster genes and samples to detect co-expressed genes under different subsets of conditions (Cheng and Church, 2000). Each module of genes or bicluster could contain co-expressed genes under different subsets of conditions, and genes may be contained in multiple modules. The application of biclustering to identify conserved and unique gene expression patterns across different species has been limited (Kacmarczyk et al., 2011; Waltman, 2012; Huang et al., 2019).

COMODO uses adaptive co-clustering to compare up to three species (Zarrineh et al., 2010, 2014). The algorithm starts with a gene–gene correlation matrix where each axis of the matrix is for one of two species, and genes that are co-expressed more highly are grouped together in modules at a specified threshold, which is determined using biclustering (Bergmann et al., 2003). The groups below the diagonal entries in the matrix that are locally more co-expressed with each other than with their neighboring genes are considered the seed modules. These seeds are expanded in each species until a pair of modules is obtained for which the number of shared orthologs is statistically optimal relative to the size of the modules. Module seeds linked by a sufficient number of orthologous gene pairs are gradually extended by traversing the space of possible cluster threshold combinations, using a combination of greedy and brute force search, represented on the gene–gene threshold matrices of each species until optimality is reached. These comparison techniques appear to have several drawbacks. First, the method of evaluation relies on the quality of functional annotation available for each species. Also, multiple cut-offs may need to be applied to determine the best co-expression stringency values for identifying possible seed modules. Lastly the researchers explain that the species they compare have genes that have one or two corresponding homologs in the other species, which is required for their method to work as expected (Zarrineh et al., 2014). Therefore, if the species compared are evolutionarily distant, or have a large portion of one-to-many or many-to-many mappings, using their statistic may not be possible.

Clustering and biclustering are useful strategies to reduce the dimensions of gene expression data. Both of these strategies can be used to identify modules of genes, which can be utilized for functional analyses or comparisons between the identified modules (Saha et al., 2017). Below we discuss and demonstrate one of the more common strategies used to construct and compare GCNs that utilizes clustering.

##### 5.1.1.1. WGCNA for Comparing Gene Co-expression Networks

One of the most widely used techniques used for module detection in GCNs is weighted gene co-expression network analysis (WGCNA). Although WGCNA was created in 2008, it is still commonly used to detect potentially important modules of genes associated with diseases (Allen et al., 2018; Swarup et al., 2019), biological pathways (Silva-Vignato et al., 2019), and development (Spadafora et al., 2018). First, unsigned or signed correlation is calculated using Equations (1) or (2), respectively. These values are used to construct the adjacency matrix, which is a quantitative measure of the strength of the relationship between each pair of genes. Each value of the adjacency matrix is raised to a power β, which is the smallest value of β that can be used where a scale-free topology is achieved. Next, WGCNA uses a topological overlap measure (TOM), which is a combination of the adjacency value between a pair of genes as well as the adjacency values these genes have with other genes to which they are connected.

WGCNAis also utilized for its module conservation statistics to make comparisons across modules of different clusterings and species (Du et al., 2020; Pembroke et al., 2021). In order to measure the preservation of a module, WGCNA can be used to determine if it is reproducible (or preserved) in an independent test network. One score is Zsummary score, which is a composite score of density and connectivity preservation statistics to determine if a module is significantly more similar to a reference module than a random sample of genes (Langfelder et al., 2011). As module size dependence could be an issue, medianRank can also be calculated for each module, which is a rank-based measure of the density and connectivity statistics. Each module is ranked based on the observed values for the statistics for each module.

**Figure 4:**
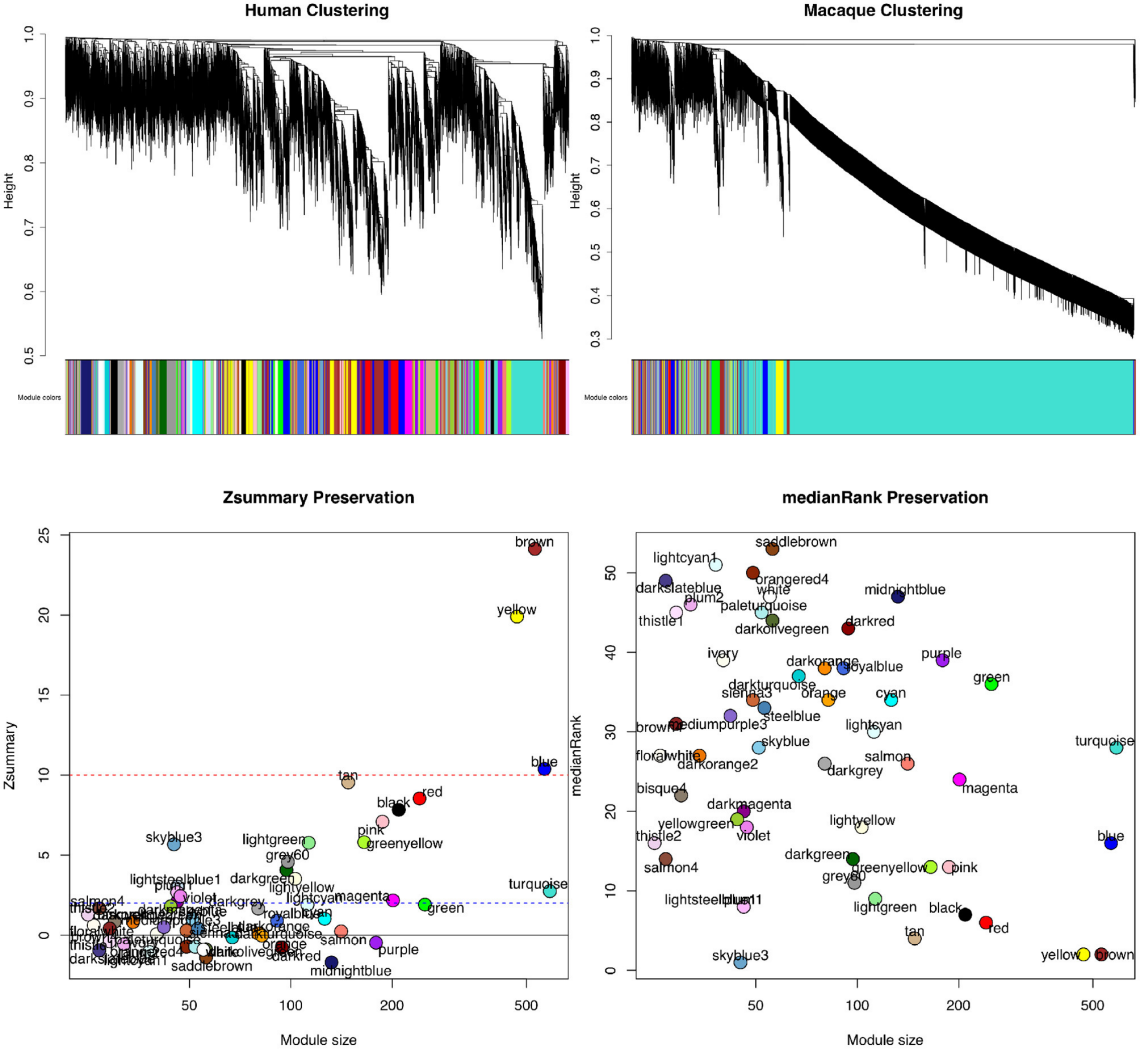
Module preservation statistics comparing signed gene co-expression networks constructed using prefrontal cortex samples from human and macaque. To generate the networks, a power of β = 8 for soft thresholding was applied to create a scale-free network topology. The clustering merge height was set to 0.20 to generate the clusters shown in the dendrogram and module color images **(top)**. The minimum module size allowed was 30 genes. From both Zsummary **(bottom left)** and medianRank **(bottom right)** preservation scores using mouse as the reference network, the most preservation is observed for the yellow and brown modules. A Zsummary score >2, but <10 indicates moderate preservation, while a score greater than 10 indicates strong module preservation. A low score for medianRank indicates high module preservation. One limitation of Zsummary score is that it often shows a dependence on module size meaning larger modules tend to get a higher score. It is also computationally intensive as it relies on permutation tests to determine significance. Although medianRank is not module size dependent like Zsummary, one drawback of medianRank is that it is rank based and therefore, it can only measure relative preservation. For example, the yellow and brown module with low medianRank scores may not be that well preserved, but it is the most preserved in comparison to the other modules discovered.

Figure 4 shows the results of WGCNA applied to publicly available RNA-seq datasets in human and macaque from Bozek et al. (2014). Samples from the prefrontal cortex were used to construct the GCNs. Relatedness of network connectivity to molecular rates of evolutions has also been studied by using WGCNA and estimates of dN (nonsynonymous substitutions per site) and dS (synonymous substitutions per site) (Masalia et al., 2017; Mack et al., 2019). More highly connected genes or genes found in a greater number of cross-tissue modules showed greater sequence constraint.

Network alignment alternatively tries to find the node correspondence between networks that leads to highly similar conserved network regions. Both approaches have their own set of challenges and depending on the biological question of interest, and how well characterized a species is, one approach may have advantages over the other.

### 5.2. Alignment-Based Methods and Applications to Gene Co-expression Networks

Table 1 shows GCN alignments that have been published in literature. Few graph alignment methods have been described from a GCN perspective or utilized to compare GCNs across different species to make inferences about their evolution.

**Table 1:** Studies utilizing an alignment strategy to compare GCNs.

**Organism**	**References**	**Method/Technique**	**Sample description**	**Alignment type**
Rice	Ficklin and Feltus (2011)	IsoRankN	508 microarray samples	Global, pairwise/multiple
Maize	Ficklin and Feltus (2011)	IsoRankN	253 microarray samples	Global, pairwise/multiple
Arabidopsis	Wang et al. (2013)	Subnetwork alignment	Leaf, flower, shoot microarray samples	Local, pairwise
Poplar	Wang et al. (2013)	Subnetwork alignment	Leaf, flower, shoot microarray samples	Local, pairwise
Mouse	Berg and Lässig (2006)	Bayesian alignment	61 tissues	Global, pairwise
Human	Berg and Lässig (2006)	Bayesian alignment	79 tissues	Global, pairwise
Mouse	Wang et al. (2009)	SCHype	300 microarray liver samples	Global/local, pairwise
Human	Wang et al. (2009)	SCHype	423 microarray liver samples	Global/local, pairwise
Rat	Wang et al. (2009)	SCHype	382 microarray liver samples	Global/local, pairwise
Mouse	Towfic et al. (2010)	BiNA	45 tissues, organs, and cell lines, 90 microarray samples	Local, pairwise
Pig	Towfic et al. (2010)	BiNA	16 tissues, 64 microarray samples	Local, pairwise
Human	Towfic et al. (2010)	BiNA	46 tissues, organs, and cell lines, 85 microarray samples	Local, pairwise
Mouse	Towfic et al. (2012)	BiNA	33 ligands, 422 microarray B-cell samples	Local, pairwise
Fly	Yan et al. (2014)	IsoRank	30 developmental stages RNA-seq samples	Global, pairwise
Worm	Yan et al. (2014)	IsoRank	33 developmental stages RNA-seq samples	Global, pairwise
Fly	Nguyen et al. (2019)	ManiNetCluster	12 timepoints RNA-seq samples	Global, pairwise
Worm	Nguyen et al. (2019)	ManiNetCluster	25 development stages RNA-seq samples	Global, pairwise
Human	Ovens et al. (2021)	Juxtapose	12 RNA-seq samples per species	Global/local, pairwise
Chimpanzee	Ovens et al. (2021)	Juxtapose	12 RNA-seq samples per species	Global/local, pairwise
Macaque	Ovens et al. (2021)	Juxtapose	12 RNA-seq samples per species	Global/local, pairwise
Mouse	Ovens et al. (2021)	Juxtapose	12 RNA-seq samples per species	Global/local, pairwise

The alignments presented in Table 1 utilize local or global measures of similarity, or a combination of both strategies, for different evolutionary applications. Many alignments also focused on detecting potential evidence of conservation based on topological and biological similarity (Berg and Lässig, 2006; Wang et al., 2013; Nguyen et al., 2019). Some methods were applied for functional annotation transfer (Towfic et al., 2010, 2012; Ficklin and Feltus, 2011; Michoel and Nachtergaele, 2012). After alignments were made, similarities between networks such as similar network centralities and conserved hub genes were studied as well as likely conserved biological pathways. Others focused on measures of global and local similarity that reflected known biology and evolutionary relationships after the alignment of the networks (Ovens et al., 2021). More details on the results of each method can be found in the Appendix. It should be noted that not all of the applications of network alignment to the study of evolution have been thoroughly explored by these studies and there is still much that could be done for future research in this area.

There are several limitations to studying evolution when applying network alignment to GCNs as presented in this section. In general, the majority of studies identified in this section only consider 2 or 3 species when utilizing alignment-based methods to compare GCNs. Therefore, it is challenging to make any inferences about the evolution of genes and the processes they drive as more than 2 species are required in order to provide evolutionary trajectory. Furthermore, identifying evidence of adaptation across GCNs is rarely the focus of alignments. As heuristics are used with the goal of identifying areas of conservation in the networks, it may not imply that what is not identified as conserved should be considered evidence of adaptation. These methods have also not been systematically compared with other network comparison strategies so it is not clear to what effect aligning the networks has on detecting evidence of conservation or adaptation. From Table 1, it is also clear that RNA-seq has not been highly utilized to perform network alignments although there are many instances of RNA-seq being used to construct, analyse, and evaluate GCNs in other ways (Iancu et al., 2012; Ballouz et al., 2015).

Utilizing network alignment methods to study evolution with GCNs comes with several technical challenges as well. In the following section, characteristics of GCNs that may prevent them from being utilized to study evolution using network alignment are discussed as well as describing potential areas of future research in the area.

## 6. Limitations, Challenges, and Future Directions

Using gene co-expression data for network analysis and alignment has some advantages over PPI network analysis and alignment, such as the much larger availability of data for the study of transcriptomics, but it also has some limitations. Gene co-expression cannot provide a full understanding of complex gene-gene interactions because they cannot distinguish between direct and indirect interactions. In other words, if they are viewed as networks that only contain direct, causative, and directional interactions, GCNs can contain many false positive interactions and the interpretation of evolutionary rewiring is more limited. Using large numbers of samples may reduce the number of false positive edges in GCNs, but depending on the thresholds used to decide what edges should be included, there is still a large number of edges to consider. False positive or false negative interactions may also be observed (or not observed) due to technical artifacts, including poor experimental design, incorrect data preprocessing, and inappropriate contrast methods (Parsana et al., 2019). This is why co-expression network analysis tends to focus on changes that are occurring in groups or modules of genes. GCN network alignment is an under-utilized tool for identifying conserved subnetworks across multiple species to study evolution.

The sign of the edge weights connecting nodes of a network can mean different things depending on whether the network is a PPI network or GCN. How each network alignment algorithm handles weighted networks may have implications as to the components of the networks the methods identify as being considered conserved or species-specific. Depending on how often differences in the edge weight sign are observed when comparing the relationship between genes in two species, it may be important, or negligible. One possibility to determine if a method is appropriate without modification for GCN alignment would be to have a network with all positively correlated genes and gradually incorporate negatively weighted genes into the network until there are modules of positively correlated genes that are negatively correlated with other module(s). How the methods align these networks respond to changes in the edge weights may provide an indication of whether it would be informative when using a network with important negative correlations.

Another possible limitation is the sample size of the dataset used to construct a network (Ballouz et al., 2015; Ovens et al., 2020) and finding multi-species studies to make evolutionary inferences. As it is often impractical to expect large datasets to be generated containing many species, it would be beneficial to make use of other publicly available datasets. However, this can result in technical challenges where network structure is determined in part by data biases. Although batch normalization methods are available, there are few normalization methods to address differences between environmental conditions (Nygaard et al., 2016). For example, not all species may be sequenced by the same lab or have different conditions in which they are raised and bred. Therefore,a comparative method to uniformly analyze cross-condition or cross-species gene expression data is essential. Further, the potential construction and comparison of sample-specific GCNs from single transcriptomic profiles may offer new insights into network evolution and better understand sample-specific differences (Kuijjer et al., 2019; Jahagirdar and Saccenti, 2020).

Since graph alignment, in general, has been utilized for so long (Clark and Kalita, 2014; Faisal et al., 2015; Elmsallati et al., 2016; Emmert-Streib et al., 2016; Meng et al., 2016b; Guzzi and Milenković, 2017), application of more of these methods to GCNs may be a good first step before attempting to create new alignment methods specifically for GCNs. PPI networks, for example, have been utilizing methods to align tagged social networks (Michoel and Nachtergaele, 2012; Zhang and Philip, 2015). At the very least, GCN aligners should be systematically compared to other PPI alignment methods to show how they are suited for this task. As IsoRank has not performed very well in PPI network alignment based on evaluation studies (Malod-Dognin et al., 2017), it may be beneficial to adopt others that have performed better to make alignments in the future.

Multilayer networks can provide better modeling for complex networks like biological networks. However, their usage is still new in some domains, including GCNs, but commonly applied in other research areas such as epidemiology (Hammoud and Kramer, 2020). These networks are node-aligned networks with layers representing co-expression profiles in different settings, including different time points, diseases, cells, or organisms (Rai et al., 2017). The layers could also be across networks from different domains such as DNA, protein, and metabolite layers (Klosik et al., 2017). Using network alignment in order to either construct or compare these networks while incorporating GCNs may be helpful. However, limitations may be encountered when dealing with large and complex (dense) networks in terms of visualizing these networks (Hammoud and Kramer, 2018) as well as evaluating alignments over various domains. Evaluating alignments may require biological expertise and experimental validation over various domains depending on the data used to make the multilayer network.

Finally, as GCN structure tends to be difficult to compare, one possibility for future research in cross-species GCN analysis is to utilize embedding strategies, typically used in natural language processing to generate numerical representations for genes. Traditional techniques such as matrix factorization have shown promising results, as well as more recent random walk-based and neural network-based methods (Grover and Leskovec, 2016). Embeddings are frequently faster than other options that operate on the original networks and are less sensitive to structural noise compared to spectral methods (Trung et al., 2020). Additionally, the learned embeddings are often applicable for downstream analysis by direct interpretation of the embedding space. Co-expression networks have recently been used to generate gene representations for single networks (Choi et al., 2018; Choy et al., 2018; Du et al., 2019) as well as multiple networks (Ovens et al., 2021), and a manifold learning technique has been used to compare co-expression networks (Nguyen et al., 2019). This may be an avenue of research for comparing an increasing number of biological networks in the future with improved and state-of-the-art techniques now available for embedding in natural language processing research.

## 7. Conclusion

Methods to compare gene expression among species include GCN alignment, which can identify quantitative evidence of adaptation or constraint acting on various groups of genes among species. The techniques used to align biological networks and infer ancestral networks are continually being improved upon to increase the agreement between topological and homology measures of network similarity.

Graph alignment techniques have been available for a long time and used for many different applications, so we reviewed how network alignment has been applied to GCNs, highlighting any crossover with PPI alignment techniques. As the alignment of GCNs becomes increasingly common, other research areas outside of biological research might provide insights. New network comparison techniques should be enlisted to compare GCNs in more organisms with the increase in transcriptomic data from newer high-throughput technologies.

## Author Contributions

KO wrote the paper and generated the figures. BE and IM co-supervised the work and assisted with revision of the manuscript. All authors contributed to the article and approved the submitted version.

## Conflict of Interest

The authors declare that the research was conducted in the absence of any commercial or financial relationships that could be construed as a potential conflict of interest.

## Publisher's Note

All claims expressed in this article are solely those of the authors and do not necessarily represent those of their affiliated organizations, or those of the publisher, the editors and the reviewers. Any product that may be evaluated in this article, or claim that may be made by its manufacturer, is not guaranteed or endorsed by the publisher.

## Appendix

The following descriptions provide additional details for the methods listed in Table 1 in section 5.2.

Ficklin and Feltus (2011) utilized IsoRankN (Liao et al., 2009), designed for PPI network alignment, to compare GCNs constructed from rice and maize. The focus was to transfer functional annotation from maize to the less-characterized rice GCN. They identified 194 genes that had unknown function in rice through 3,092 conserved edges, which suggested associated biological processes such as seed storage. Interestingly, although sequence orthology in general is a common strategy for transferring functional annotation from one species to another, the cost function used to generate the alignment between these species was weighted with more emphasis toward topological similarity. This suggests that similarities in topological structure between GCNs is informative for functional annotation transfer.

A study of *Arabidopsis* and *Poplar* incorporated analysis of network topology and also went so far as to align the networks to identify the conserved and species-specific functions of cell-wall related genes (Wang et al., 2013). Subnetworks associated with cell wall genes in leaf, flower, and shoot tissues between the two plant species were aligned while considering the neighboring orthologous genes. Tissues that had good alignments were considered to likely have more conserved function. They also separately investigated network centralities including clustering coefficient and eigenvector centrality for measuring a gene's global influence over the entire network. Conserved hub genes and tissue-specific hub genes across networks were discovered.

Berg and Lässig (2006) utilized a probabilistic alignment procedure for biological network alignment based on their edge and node similarity and attempted to maximize their proposed score based on a mapping to a generalized quadratic assignment problem. This was another method proposed to identify conserved modules of genes, which in this case was applied to compare human and mouse GCNs. However, they applied their method to a limited number of genes considered housekeeping genes that were expressed in all samples and showed a low variance of expression levels across samples in both species, as well as genes with a high expression similarity with at least one of the genes considered housekeeping genes. Furthermore, although they claim to analyze the evolution of GCNs between humans and mice, studying evolution is challenging given that only two species were compared.

SCHype addresses local and global alignment with recursive spectral clustering and biclustering algorithms of hypergraphs (generalizations of graphs where the edges can exist between arbitrary subsets of nodes, rather than just two) to identify sets of nodes in each species with a greater than expected number of conserved interactions (based on co-expression in this case) between them (Michoel and Nachtergaele, 2012). The technique is used to discover densely interconnected genes by computing the dominant eigenvector and then converting it to a discrete set of vertices. Uniqueness of the dominant eigenvector guarantees unambiguity of the solution and rapid convergence of the procedure and allows for analysis of complex homology groups, unlike the potential drawbacks of using Pearson's chi squared test from COMODO (Zarrineh et al., 2010). Again, this is another technique that has been applied to GCNs for the purpose of functional annotation transfer (Reyes et al., 2017). Importantly, individual experiments from the series did not cluster together in SCHype clusters. This could indicate different normalization techniques may be required to account for these differences to make better comparisons across experiments.

BiNA is another PPI network alignment method that has been applied to co-expression data, and works by breaking down the networks being compared into subgraphs to align each of them (Towfic et al., 2010, 2012). A “*k*-hop subgraph” for each gene *g* is constructed by including any gene that can be reached from *g* within *k* edges. Each of the subgraphs in one co-expression network is compared to the subnetworks in the second network to find the best match based on a similarity measure, taking into account the sequence homology of the genes as well as the general topology of the neighborhood around each gene. The authors used their method for ortholog detection (Towfic et al., 2010), and to identify B-cell ligand processing pathways (Towfic et al., 2012). None of the similarity measures employed by this method have utilized weighted edges, but there is potential to extend the method to handle weighted graph scenarios.

ManiNetCluster is a recent strategy for alignment that projects two GCNs into a common lower dimensional space on which the Euclidean distances between genes preserve the geodesic distances between them (Nguyen et al., 2019). This distance was used as a metric to detect manifolds embedded in the original high-dimensional network space. This is different from the other alignment methods covered in that it is a subspace learning approach, embedding the nodes across different networks into a common low dimensional representation. The network representations were used to form a multilayer network that could be clustered to a number of cross-network gene modules. The benefit of such a method is that only the networks are required to learn the underlying structure of the data. This method was only tested on a cross-species comparison using 1,882 fly genes and 1,925 worm genes. Therefore, it would need to be tested on a much larger network with more species to determine its utility for making evolutionary inferences.
